# Altered renal sodium handling and risk of incident hypertension: Results of the Olivetti Heart Study

**DOI:** 10.1371/journal.pone.0171973

**Published:** 2017-02-14

**Authors:** Lanfranco D’Elia, Francesco P. Cappuccio, Roberto Iacone, Ornella Russo, Ferruccio Galletti, Pasquale Strazzullo

**Affiliations:** 1 Department of Clinical Medicine and Surgery, ESH Excellence Center of Hypertension, “Federico II” University of Naples Medical School, Naples, Italy; 2 University of Warwick, Division of Health Sciences, WHO Collaborating Centre for Nutrition, Coventry, United Kingdom; Emory University Department of Medicine, UNITED STATES

## Abstract

Renal tubular sodium (Na) handling plays a key role in blood pressure (BP) regulation. Several cross-sectional studies reported a positive association between higher proximal tubule fractional reabsorption of Na and BP, but no prospective investigation has been reported of this possible association. Hence, the purpose of this study was to estimate the predictive role of renal Na handling on the risk of incident hypertension and the changes in BP occurring in the 8-year follow-up observation of a sample of initially normotensive men (The Olivetti Heart Study). The study included 294 untreated normotensive non-diabetic men with normal renal function examined twice (1994–95 and 2002–04). Renal tubular Na handling was estimated by exogenous lithium clearance. Fractional reabsorption of Na in proximal and distal tubules was calculated and included in the analysis. At baseline, there was no association between BP and either proximal or distal fractional reabsorption of Na. At the end of the 8-year follow-up, direct associations were observed between baseline proximal (but not distal) Na fractional reabsorption and the changes occurred in systolic and diastolic BP over time (+2.79 and +1.53 mmHg, respectively, per 1SD difference in proximal Na-FR; p<0.01). Also multivariable analysis showed a direct association between baseline proximal Na fractional reabsorption and risk of incident hypertension, independently of potential confounders (OR: 1.34, 95%CI:1.06–1.70). The results of this prospective investigation strongly suggest a causal relationship between an enhanced rate of Na reabsorption in the proximal tubule and the risk of incident hypertension in initially normotensive men.

## Introduction

The role of the kidney in blood pressure (BP) regulation was perceived since the early ‘40s [1] and comprehensively described over the last decades of the past century [2,3]. In this general context, particular attention has been paid to renal tubular sodium handling [4]. Alterations in sodium handling underlie several forms of monogenic hypertension [5,6] but have been described as well in patients with salt-sensitive essential hypertension carrying particular allelic variants of genes encoding for ion transport molecules in the tubular epithelium [7–10]. Furthermore, higher proximal tubular sodium reabsorption estimated by the clearance of endogenous or exogenous lithium has been found in individuals with abdominal adiposity [11], diabetes [12] and metabolic syndrome [13,14], conditions in turn predisposing to high BP and featuring an altered pressure-natriuresis relationship [15]. There is however no prospective investigation testing the hypothesis that renal tubular sodium handling in clinically healthy normotensive individuals is associated with larger increases in BP and with a greater risk to develop hypertension over time. We tested this hypothesis in the initially normotensive participants of the Olivetti Heart Study, a prospective investigation of the metabolic, nutritional and genetic determinants of cardiovascular disease involving the male workforce of the Olivetti factories in Campania, Southern Italy. The study sample was made of 294 untreated normotensive non-diabetic men with normal renal function examined twice at 8 year distance (1994–95 and 2002–04). Renal tubular Na handling was estimated by the clearance of exogenous lithium.

## Results

The relevant characteristics of the study participants at baseline are reported in Table 1. The analysis of the possible relationships between the participants’ most relevant characteristics at baseline showed an inverse and significant association between proximal and distal Na reabsorption (r = -0.26, p<0.01), a direct association between distal Na reabsorption and creatinine clearance (r = 0.18, p<0.01), while no association was detected between proximal Na reabsorption and creatinine clearance (p>0.05).

**Table 1:** Baseline characteristics of the study participants.

N. of participants	294
Proximal fractional Na reabsorption (%)	74.6 (6.7)
Distal fractional Na reabsorption (%)	95.0 (1.8)
Age (yrs)	49.0 (6.6)
BMI (kg/m^2^)	26.4 (2.8)
Overweight (%)	60
Obesity (%)	10
Systolic BP (mmHg)	119.6 (9.5)
Diastolic BP (mmHg)	78.9 (6.3)
Abdominal Circumference (cm)	92.6 (8.1)
Central obesity (%)	10
Creatinine Clearance (mL/min/1.73 m^2^)	91.3 (17.9)
Homa Index (Unit) ^1^	1.74 (1.66)
Insulin resistance (%)	54
Hypercholesterolemia (%)	70
Hypertriglyceridemia (%)	37
Physical Activity (%)	28
Alcoholic beverages (%)	80
Cigarette Smoking (%)	48
Estimated sodium intake (mmol/day)^2^	183.8 (30.7)

Data are expressed as means (SD), or as percentages;

BP, Blood Pressure;

^1^ Geometric mean;

^2^Calculated by Tanaka’s formula.

Table 2 shows the changes in BP and other relevant variables observed at the 8-year follow-up examination as compared with baseline. In the sample as a whole, there was a significant overall trend to increased BMI and abdominal adiposity as indicated by greater waist circumference. A decline in renal function as estimated by creatinine clearance was also detected. BP increased in the whole sample and its changes were directly and significantly associated with baseline proximal Na reabsorption whereas no such association was seen with distal Na reabsorption.

**Table 2:** Eight year changes in the participants’ main characteristics and their correlation with baseline fractional reabsorption of sodium at the proximal and distal tubular level.

	Δ	Proximal Na reabsorption	Distal Na reabsorption
Δ BMI (kg/m^2^)	0.4±1.7^1^	r = 0.08	r = -0.10
Δ SBP (mm Hg)	13.6±13.8^1^	r = 0.20^1^	r = -0.02
Δ DBP (mm Hg)	8.6±9.1^1^	r = 0.17^1^	r = -0.01
Δ Abdominal Circumference (cm)	3.8±5.8^1^	r = -0.01	r = -0.01
Δ Creatinine Clearance (mL/min/1.73 m^2^)	-3.5±25.4^1^	r = -0.03	r = -0.05
Δ Homa Index (Unit)	0.1±1.7	r = 0.06	r = -0.13

Data are expressed as means ±SD; Δ, changes after 8 years were calculated as final minus basal measurements; SBP, systolic blood pressure; DBP, diastolic blood pressure.

^1^ p –value < 0.01.

**Fig 1:**
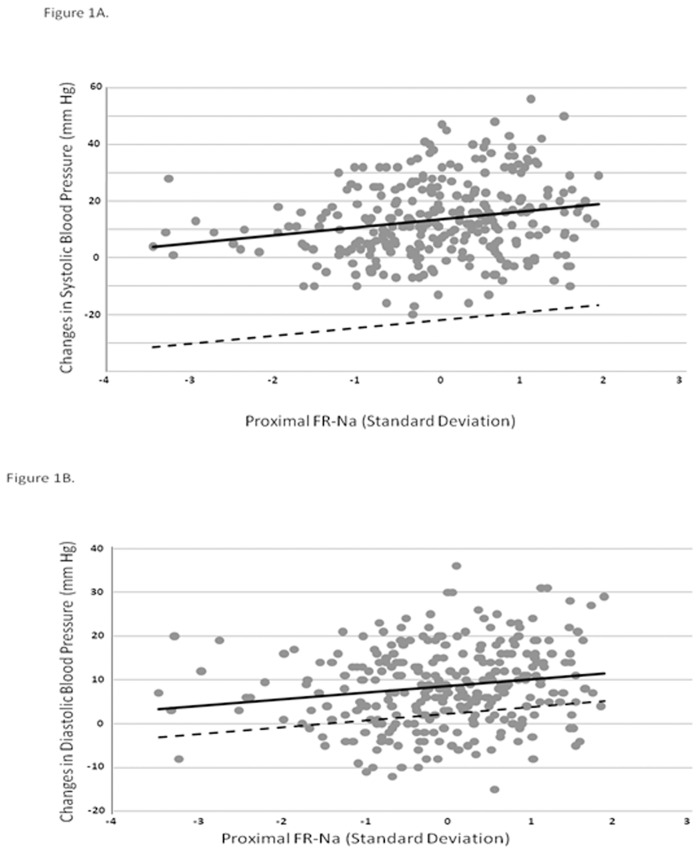
Changes in systolic (A) or diastolic (B) blood pressure and fractional proximal reabsorption of sodium (proximal FR-Na), after 8 years of follow-up. Proximal FR-Na expressed as changes in standard deviation (SD) (1SD = 6.7%). Solid line: model unadjusted (p< 0.01). Dash line: model adjusted for baseline age, BMI, systolic or diastolic blood pressure (rank), physical activity, alcohol intake, cigarette smoking, insulin sensitivity, and antihypertensive therapy at follow-up (p = 0.01).

Table 3 provides the results of linear regression analyses of the changes observed in BP as a function of baseline proximal and distal Na reabsorption. This approach confirmed the significant association between proximal tubular sodium reabsorption and the increase in BP over time: this association remained statistically significant in multivariable analyses, accounting for potential confounders including baseline BMI or changes in BMI over the 8-year observation period (Fig 1) (S1 Table). In addition, linear regression analyses without participants on antihypertensive treatment (n = 36) confirmed the positive and significant association between proximal Na reabsorption and changes in blood pressure over time (systolic BP changes: β = 2.53, 95%CI = 1.05 to 4.06, p<0.01; diastolic BP changes: β = 1.50, 95%CI = 0.49 to 2.51, p<0.01).

**Table 3:** Changes in blood pressure for 1SD difference in baseline proximal and distal fractional reabsorption of sodium (linear regression analysis).

SBP change for 1SD ↑ in Proximal Na reabsorption	β (95%CI)	p-value
Unadjusted	2.79 (1.23 to 4.34)	< 0.01
Multivariable Model 1 ^1^	2.17 (0.43 to 3.92)	0.01
Multivariable Model 2 ^2^	2.17 (0.44 to 3.89)	0.01
DBP change for 1SD ↑ in Proximal Na reabsorption		
Unadjusted	1.53 (0.50 to 2.56)	<0.01
Multivariable Model 1 ^1^	1.46 (0.33 to 2.60)	0.01
Multivariable Model 2 ^2^	1.37 (0.26 to 2.48)	0.02
SBP change for 1SD ↑ in Distal Na reabsorption		
Unadjusted	-0.35 (-1.94 to 1.24)	0.67
Multivariable Model 1^1^	-0.68 (-2.19 to 0.83)	0.37
Multivariable Model 2 ^2^	-0.33 (-1.86 to 1.19)	0.67
DBP change for 1SD ↑ in Distal Na reabsorption		
Unadjusted	-0.05 (-1.09 to 0.99)	0.92
Multivariable Model 1 ^1^	-0.28 (-1.25 to 0.69)	0.57
Multivariable Model 2 ^2^	-0.08 (-1.06 to 0.89)	0.86

SBP, systolic blood pressure; DBP, diastolic blood pressure; SD, standard deviation.

^1^ Multivariable model 1: adjusted for baseline age, **BMI**, SBP or DBP (rank), physical activity, alcohol intake, cigarette smoking, insulin sensitivity and antihypertensive therapy at follow-up.

^2^ Multivariable model 2: adjusted for baseline age, SBP or DBP (rank), physical activity, alcohol intake, cigarette smoking, insulin sensitivity, **BMI changes** and antihypertensive therapy at follow-up.

Again, no association was detected between distal Na reabsorption and BP changes over time.

The overall incidence of hypertension was 52% during the 8-year follow-up observation. No significant association was detected between the risk of incident hypertension and the participants’ habitual salt intake estimated by the Tanaka method at baseline (OR: 1.00, 95% C.I. 0.99–1.01; p = 0.65) and by 24h urine collection at follow-up examination (OR: 1.00, 95% C.I. 0.99–1.01; p = 0.69). By contrast, a linear relationship was found between proximal Na reabsorption and risk of hypertension (p for non-linearity = 0.07).

Logistic regression analysis showed a significant increased risk of hypertension for 1 SD higher value of proximal Na reabsorption (OR: 1.47; 95% C.I. 1.11–1.95; p<0.01) after adjusting for multiple confounding variables (Table 4) (S2 Table). No such association was observed with distal Na reabsorption.

**Table 4:** Eight year risk of incident hypertension for 1SD change in proximal and distal fractional reabsorption of sodium (logistic regression analysis).

	Risk of Incident Hypertension OR (95% CI)	P-value
1SD ↑ in Proximal fractional Na reabsorption		
Unadjusted	1.34 (1.06–1.70)	0.02
Multivariable Model 1 ^1^	1.46 (1.11–1.94)	<0.01
Multivariable Model 2 ^2^	1.47 (1.11–1.95)	<0.01
1SD ↑ in Distal fractional Na reabsorption		
Unadjusted	0.91 (0.73–1.15)	0.45
Multivariable Model 1 ^1^	0.90 (0.68–1.18)	0.44
Multivariable Model 2 ^2^	0.93 (0.71–1.23)	0.62

OR, Odds Ratio; SBP, systolic blood pressure; SD, standard deviation.

^1^ Adjusted for baseline age, **BMI**, SBP, physical activity, alcohol intake, cigarette smoking, insulin sensitivity.

^2^ Adjusted for baseline age, SBP, physical activity, alcohol intake, cigarette smoking, insulin sensitivity, and **BMI changes**.

After exclusion of participants on antihypertensive treatment (n = 36), logistic regression analysis again showed that a 1-SD higher proximal Na reabsorption was associated with a significantly greater risk of hypertension (OR: 1.46; 95%CI: 1.12-1-91; p<0.01).

The analysis stratified for severity of hypertension showed a significant association between the degree of BP elevation and the level of proximal Na reabsorption after exclusion of participants on antihypertensive therapy (p = 0.007) (S3 Table).

## Discussion

Following the results of previous studies by our and by other research groups, the present analysis of a selected sample of adult male population drawn from the Olivetti Heart Study database tested the hypothesis that a higher rate of proximal tubular sodium reabsorption predicts a greater risk of hypertension. Its main novel finding was indeed that a higher rate of proximal Na reabsorption at baseline was associated with larger increments in systolic and diastolic BP and with a greater probability to develop hypertension in 8 years, consistent with our hypothesis. The association remained statistically significant upon accounting for a number of measured potential confounders, including the participants’ estimated habitual dietary sodium intake. Moreover, the analysis showed a significant association between the severity of hypertension and the level of proximal tubular Na reabsorption.

Strengths of our study are its prospective design, the relatively long follow-up observation period, the inclusion of only untreated non-diabetic participants with normal BP and renal function at baseline, the use of a dependable technic for the assessment of renal tubular sodium handling, the carefully standardized direct measurement of BP and related variables at both baseline and follow-up and, in general, the careful standardization of data collection.

We also acknowledge a few limitations, however. The first one is the participation of adult white male individuals only, which makes our results only generalizable to people of male gender and of Caucasian ethnicity. Another weakness is the lack of intermediate BP measurements during the 8-year follow-up observation period with the consequent inability to perform a time-to-event analysis relative to the rate of increase in BP and to the incidence of hypertension.

The results of our study are otherwise robust and in keeping with previous knowledge of the role of the kidney in BP homeostasis. They provide further support to the hypothesis that alterations in renal tubular sodium handling play a causal role in the development of so-called essential hypertension.

This hypothesis was originally made upon detection of monogenic forms of hypertension in which the mutation in a single gene encoding for one of several proteins involved in sodium and water transport across the tubular epithelium provoked a volume-dependent increase in BP [6,16–18], which was responsive to reduced sodium intake and to the use of diuretics. Although these alterations are rare (less than 1% of all prevalent hypertension), the exact definition of the mechanisms leading to the increase in pressure in these conditions was strongly supportive of the importance of renal tubular sodium handling in hypertension.

Later on, it has become clear that not only rare genetic mutations but also several relatively common genetic variants are associated with both altered renal tubular sodium handling and increased susceptibility to hypertension [7–10]. Examples are given by the Gly460Trp variant of the α-adducin gene and by the Arg40Ser variant of the glucagon receptor gene which are associated with an enhanced rate of proximal tubular sodium reabsorption [7,10]. Even more important, in addition to genetic alterations, largely prevalent metabolic and neuro-endocrine conditions such as obesity and elevated sympathetic tone, known to increase susceptibility to hypertension, also were found to affect the renal tubular sodium and water handling [11,15,19].

An increased rate of proximal Na reabsorption has been linked to sodium-sensitivity of BP in two specific studies. In one of these, a small sample of normotensive individuals was repeatedly evaluated while on a sodium restricted (70 mmol sodium/day) or on the habitual sodium-rich diet (185 mmol sodium/day) [20]. Proximal Na reabsorption was related to salt intake being significantly reduced in the group as a whole while on high salt intake. However, when the participants were stratified in 3 groups according to the degree of BP response to changing sodium intake, the subjects whose BP increased most on high sodium intake had the least reduction in proximal Na reabsorption, whereas distal Na reabsorption on high sodium intake decreased to a similar level in all 3 groups.

A similar study was performed in hypertensive patients. In this case, patients whose BP was most sensitive to sodium intake showed a paradoxical increase, rather than a decrease, in proximal Na reabsorption upon salt intake elevation, again with no differences in distal Na handling [21].

In a subsequent prospective assessment of the risk of incident hypertension in a small sample of 36 individuals participating in our original study, it was found that those who had shown the highest degree of salt-sensitivity at baseline had a significantly greater risk of incident hypertension [22].

It may be of concern that the results of the present study did not indicate a relationship between the participants’ estimated salt intake and risk of hypertension. However, it must be considered that both the Tanaka method based on a single fasting urine specimen and the information provided by a single 24h urine collection result in very inaccurate estimates of the habitual sodium intake at the individual level [23].

In any case, it would be hard to imagine an influence of excess salt intake on the observed direct relationship between proximal Na reabsorption and risk of hypertension in as much as a higher salt intake is physiologically associated with a lower not higher rate of proximal tubule sodium reabsorption, as we also showed in our above mentioned study [20].

The present report is the first one directly relating an altered renal tubular reabsorption of sodium to the risk of incident hypertension in a relatively large sample of individuals who were normotensive and clinically healthy at baseline and were observed for a reasonably long period.

The results of our study add to the evidence accumulated through previous studies in favour of a causal role of alterations in renal sodium handling in the trend to BP increase and in the risk to develop hypertension over time. Given the demonstrated link between altered renal sodium handling and salt-sensitivity of BP, these results also provide strong support to WHO recommendations to reduce salt intake to prevent hypertension and its cardiovascular and renal complications [24].

## Materials and methods

### Study population

The Olivetti Heart Study was an occupational investigation of the male workforce of the Olivetti factories in Southern Italy (Pozzuoli-Naples and Marcianise-Caserta), as previously described [25]. A total of 1,085 individuals (95% of the total male workforce) aged 25–75 years were examined in 1994–95. For the purposes of the present analysis, we excluded participants who were diagnosed as hypertensive at baseline (BP ≥ 140/90 mmHg or on current antihypertensive therapy prescribed by the participant’s personal physician; n = 465). We also excluded participants with clinical evidence of renal dysfunction (creatinine clearance <60 mL/min/1.73 m^2^) (n = 135), diabetes mellitus (fasting blood glucose level ≥126 mg/dL or current anti-diabetic therapy) (n = 24), individuals with missing assessment of renal sodium handling (n = 109) and participants whose demographic and anthropometric characteristics and/or cardio-metabolic risk factors were not available at baseline (n = 7). Finally, 339 clinically healthy and initially normotensive individuals were included. Of these participants, 294 (87%) were seen again in 2002–04 and were considered eligible for the present analysis. The local Ethical Committee (The Ethical Committee-Federico II University of Naples) approved the Olivetti study protocol, and the participants provided their informed written consent to participate.

### Study protocol

The OHS study procedures have been repeatedly described [25]. At both baseline and follow-up visit, physical examinations were performed between 08:00 and 11:00 h, in a quiet and comfortable room, with the participants having fasted for at least 13 h. The participants were allowed to pursue their normal activities but were discouraged from engaging in vigorous exercise. They were asked to abstain from smoking and drinking alcohol, coffee, tea and other beverages containing caffeine starting on the night before the visit.

The baseline visit included a physical examination and anthropometric measurements, a blood test, a fasting timed urine collection and the administration of a questionnaire including information on medical history, working and leisure time physical activity, smoking habit and alcohol consumption.

Systolic and diastolic BP (phase V) were measured three times, 2 min apart, with a random zero sphygmomanometer (Gelman Hawksley Ltd., Sussex, UK) after the subject had been sitting for at least 10 min. The average of the second and third reading was recorded. The diagnosis of incident hypertension during the 8-year follow-up period was defined as systolic BP ≥ 140 and/or diastolic BP ≥90 mmHg or current antihypertensive drug treatment [26].

Body weight, height and abdominal circumference were measured as described [11]. Overweight was defined as a BMI ≥ 25 kg/m^2^ and obesity as a BMI ≥ 30 kg/m^2^. Central obesity was given by an abdominal circumference value ≥ 102 cm [26].

A fasting venous blood sample was obtained for the determination of serum glucose, insulin, lipids, and creatinine. Blood specimens were immediately centrifuged and stored at –70°C until analysis. Serum total cholesterol, triglyceride, and glucose levels were measured with automated methods (Cobas-Mira; Roche, Milan, Italy). Serum insulin concentration was measured by radioimmunoassay (Insulina Lisophase; Technogenetics, Milan, Italy). Insulin sensitivity was estimated by the homeostasis model assessment (HOMA) using the formula: fasting plasma insulin (μU/mL) x fasting plasma glucose (mmol/L)/22.5. A Homa index >1.77 was considered as a cut-off value for insulin resistence.

The habitual physical activity and the drinking and smoking habits were assessed as previously reported [27]. Hypercholesterolemia and hypertriglyceridemia were defined as described [26].

The participants’ habitual sodium intake was estimated from the morning fasting urine collection using the Tanaka method [28].

The segmental tubular sodium handling was estimated by the clearance of exogenous lithium [29]. This technique is based on the notion that, whereas sodium and water are reabsorbed at numerous sites along the nephron, the lithium ion is taken up almost exclusively at proximal tubular sites, so that the amount of substance escaping reabsorption at this level is quantitatively excreted in the urine. Since lithium is transported by the same systems driving sodium and water, an alteration in the fractional excretion of lithium argues for an alteration in the reabsorption of sodium and water at the proximal tubule. This method has been used in the majority of experimental and clinical studies of renal tubular sodium handling [4] and its accuracy and reproducibility has been well documented [29].

On the day before the visit, at 22.00 hours (3-hour after the evening meal) a 300 mg lithium carbonate capsule, delivering 8.1 mmol of elemental lithium, was taken with 400 mL of tap water by the subjects. On the morning of the study, a 4-hour urine collection was obtained (from 07.00 to 11.00 hours) and, at the mid-point of the collection, a blood sample was collected. The subjects were required to remain fastedovernight and until completion of the study. Time and volume of urinary collections were recorded and a specimen was used for the analysis. Creatinine, sodium and lithium in serum were measured by the picric acid colorimetric method and in urine samples by atomic absorption spectrophotometry, and were used to estimate the renal clearance of each substance [11,30]. The fractional excretion of sodium and lithium were calculated as the ratio of sodium or lithium clearance and creatinine clearance and were used in the analysis thus neutralizing the confounding effects of age, body mass and possibly incomplete urine collections on the evaluation of segmental tubular sodium handling. These values were eventually used to calculate the fractional reabsorption of sodium at the proximal and distal tubular level, according to standard formulae [31].

A methodological assessment performed in our laboratory provided intra- and interindividual coefficients of variation of 8.5% and 14.7%, respectively, with a ratio of intraindividual to interindividual variance of 0.33 [31].

At the 8-year follow-up visit, in addition to the physical examination and to the measurement of anthropometric indexes and BP using the same procedure as at baseline, a 24-hour urine collection was obtained to estimate again the participants’ current habitual dietary sodium intake.

### Statistical analysis

All statistical analyses were performed using the SPSS software, version 15 (SPSS inc, Chicago, Ill) and the STATA Corp. software (version11.2). Since the distribution of HOMA index was skewed, our analyses were based on log-transformed values. Changes (Δ) in the participants’ main characteristics were calculated as final minus basal measurements.

Bivariate relationships between relevant participants’ features and proximal or distal Na reabsorption were evaluated by Pearson correlation analysis, both at baseline and at follow-up. Paired t-tests were used to assess differences between baseline and follow-up visit in any of the variables under investigation. Analysis of variance (ANOVA) was used to assess differences in proximal Na reabsorption among group means upon stratification for BP levels at follow-up [26]. Multivariable linear regression analysis was used to determine the independent effect of renal sodium handling on BP, adjusting for the main potential confounders. Binary logistic regression analysis was used to estimate the role of renal sodium handling on the incidence of hypertension, adjusting for confounders. In multivariable analyses of the BP changes over time, to account for the effect of baseline BP, we introduced as covariates the rank values of baseline systolic BP and diastolic BP. Potential non-linear associations between proximal (or distal) Na reabsorption and risk of hypertension were tested, with p-values < 0.05 denoting significant non-linearity [32].

The results were reported, as appropriate, as mean ± SD or SE or as percentages or as odds ratio (OR) and 95% confidence interval (CI). Two-sided P values below 0.05 were considered statistically significant.

## Supporting information

S1 Table Changes in blood pressure for 1SD difference in fractional reabsorption of sodium at the proximal tubular level (linear regression analysis). (DOC)

S2 Table Eight year risk of incident hypertension for 1SD difference in fractional reabsorption of sodium at the proximal tubular level (logistic regression analysis). (DOC)

S3 Table Fractional reabsorption of sodium at the proximal tubular level stratified for severity of hypertension, after exclusion of participants in antihypertensive treatment. (DOC)
