# Supplementary material for: Altered renal sodium handling and risk of incident hypertension: Results of the Olivetti Heart Study
Source: PLoS One. 2017 Feb 14;12(2):e0171973. doi: 10.1371/journal.pone.0171973 (PMC5308782; doi:10.1371/journal.pone.0171973)
Supplement: S1 Table — (DOC) [file pone.0171973.s001.doc]

**S1 Table. Changes in blood pressure for 1SD difference in fractional reabsorption of sodium at the proximal tubular level (linear regression analysis).**

| **SBP change** | | **β** (95%CI) | **p-value** | **t** |
| --- | --- | --- | --- | --- |
| **1SD ↑ in Proximal Na reabsorption** | | **2.17 (0.43 to 3.92)** | **0.015** | **2.46** |
| SBP (**↑** rank) | | -0.60 (-0.08 to -0.04) | <0.001 | -6.83 |
| Age (1 year) | | 0.65 (0.36 to 0.94) | <0.001 | 4.37 |
| BMI (1 Unit) | | 0.70 (0.07 to 1.34) | 0.031 | 2.18 |
| Cigarette smoking (yes) | | 1.19 (-2.23 to 4.72) | 0.50 | 0.67 |
| Physical activity (yes) | | -1.99 (-5.85 to 1.87) | 0.31 | -1.02 |
| Insulin resistance (yes) | | 0.80 (-2.69 to 8.16) | 0.65 | 0.45 |
| Alcohol intake (yes) | | -0.85 (-5.49 to 3.80) | 0.72 | -0.36 |
| Antihypertensive therapy at follow-up (yes) | | 2.73 (-2.69 to 8.16) | 0.32 | 0.99 |
| R^2^= 0.25 |  | |  |  |
| **DBP change** | |  |  |  |
| **1SD ↑ in Proximal Na reabsorption** | | **1.46 (0.33 to 2.60)** | **0.012** | **2.55** |
| DBP (**↑** rank) | | -0.04 (-0.05 to -0.03) | <0.001 | -7.12 |
| Age (1 year) | | 0.13 (-0.06 to 0.32) | 0.18 | 1.34 |
| BMI (1 Unit) | | 0.28 (-0.14 to 0.70) | 0.19 | 1.32 |
| Physical activity (yes) | | -1.31 (-3.80 to 1.18) | 0.30 | -1.04 |
| Cigarette smoking (yes) | | 0.83 (-1.53 to 3.19) | 0.49 | 0.69 |
| Insulin resistance (yes) | | 0.74 (-1.53 to 3.02) | 0.52 | 0.64 |
| Alcohol intake (yes) | | -2.28 (-3.31 to 2.75) | 0.86 | -0.18 |
| Antihypertensive therapy at follow-up (yes) | | -0.41 (-3.95 to 3.14) | 0.82 | -0.23 |
| R^2^= 0.23 | |  |  |  |
|  | |  |  |  |

SBP: systolic blood pressure; DBP: diastolic blood pressure; SD: standard deviation
