# Supplementary material for: Altered renal sodium handling and risk of incident hypertension: Results of the Olivetti Heart Study
Source: PLoS One. 2017 Feb 14;12(2):e0171973. doi: 10.1371/journal.pone.0171973 (PMC5308782; doi:10.1371/journal.pone.0171973)
Supplement: S2 Table — (DOC) [file pone.0171973.s002.doc]

**S2 Table. Eight year** r**isk of incident hypertension for 1SD difference in fractional reabsorption of sodium at the proximal tubular level (logistic regression analysis).**

|  | | **Risk of Incident Hypertension**  **OR (95% CI)** | **P-value** | **Wald-test** |
| --- | --- | --- | --- | --- |
| **1SD↑in Prox. Na reabsorption** | **1.46 (1.11 – 1.94)** | | **0.008** | **7.10** |
| SBP (1 mm Hg) | 1.07 (1.03 – 1.10) | | <0.001 | 17.19 |
| BMI (1 Unit) | 1.15 (1.04 – 1.28) | | 0.007 | 7.30 |
| Cigarette smoking (yes) | 1.94 (1.12 – 3.38) | | 0.018 | 5.57 |
| Age (1 year) | 1.05 (1.01 – 1.10) | | 0.035 | 4.43 |
| Insulin resistance (yes) | 1.48 (0.86 – 2.56) | | 0.16 | 1.98 |
| Alcohol intake (yes) | 0.72 (0.36 – 1.46) | | 0.37 | 0.81 |
| Physical activity (yes) | 1.08 (0.57 – 2.05) | | 0.80 | 0.06 |
| R^2^= 0.17 |  | |  |  |

SBP: systolic blood pressure; SD: standard deviation
