# Supplementary material for: Altered renal sodium handling and risk of incident hypertension: Results of the Olivetti Heart Study
Source: PLoS One. 2017 Feb 14;12(2):e0171973. doi: 10.1371/journal.pone.0171973 (PMC5308782; doi:10.1371/journal.pone.0171973)
Supplement: S3 Table — (DOC) [file pone.0171973.s003.doc]

**S3 Table. Fractional reabsorption of sodium at the proximal tubular level stratified for severity of hypertension, after exclusion of participants in antihypertensive treatment.**

|  | | **Proximal Na reabsorption**  **Mean (SD) %** |
| --- | --- | --- |
| “Normal” BP (142) | 73.6 (7.1) | |
| Grade 1 (96) | 75.7 (6.0) | |
| Grade 2 (17) & 3 (3) | 77.8 (5.6) | |
|  | **P for trend 0.007** | |

SD: standard deviation; BP: blood pressure; Normal BP: systolic/diastolic BP <140/90 mm Hg, Grade 1: 140-159 and/or 90-99 mm Hg: Grade 2: 160-179 and/or 100-109 mm Hg; Grade 3: >180 and/or >110 mm Hg
